# Over the Counter Sale of Antibiotics at Drug Stores Found in Mizan-Aman Town, Southwest Ethiopia: A Cross-Sectional Simulated Client Visit Study

**DOI:** 10.1155/2019/3510659

**Published:** 2019-04-07

**Authors:** Getahun Damisie, Solomon Hambisa, Mohammed Yimam

**Affiliations:** Department of Pharmacy, College of Health Sciences, Mizan-Tepi University, Mizan-Teferi, Ethiopia

## Abstract

**Background:**

Antibiotics are crucial drugs, particularly in the developing world, where infectious diseases are a common cause of death. Misuse and overuse of antibiotics have driven the emergency of antibiotic-resistant bacteria, which in turn leads to a loss of efficacy of these drugs. This study aimed to assess the professional practice on OTC sale of antibiotics at community drug retail outlets found in Mizan-Aman town.

**Methods:**

A cross-sectional simulated client visit study was conducted among community drug retail outlets found in Mizan-Aman town, Southwest Ethiopia, from 14 to 28 March, 2018. Currently, there are 18 commercially licensed community drug retail outlets in Mizan-Aman town and the study was undertaken on all drug retail outlets. Each drug retail outlet was visited once by investigators who simulated inflicting clinical scenario according to simulated client method pharmacy surveys. Three different clinical scenarios were chosen and, in each of the three cases, three levels of demand were used to obtain the antibiotic. The findings of the study were entered, cleared, coded, and stored into Epi Info version 3.5.1 and exported to Statistical Package for Social Sciences (Windows version 21) and the collected data were compiled and presented as descriptive statistics using tables and figures.

**Results:**

Most, 17 (94.4%), of drug stores out of the total 18 in which all three clinical scenarios were allotted were issued antibiotics without a need of medical prescription with three different levels of demands. Antibiotics were sold without a prescription in most (94.4%) of drug stores in which a urinary tract infection clinical scenario was presented. Similarly, antibiotics were obtained without a prescription for acute diarrhea from 16 (88.9%) drug stores. With respect to sore throat simulation, antimicrobial drugs were obtained without a prescription from 14 (77.8%) drug stores. Commonly dispensed antibiotics were Metronidazole (50.0%), Ciprofloxacin (38.9%), and Amoxicillin (71.4%) for acute diarrhea, urinary tract infection, and sore throat case scenarios, respectively. Only 1 drug store (5.5%) refused to dispense any kind of antibiotics.

**Conclusion:**

The results of this study demonstrate that nonprescription sales of antibiotics were highly pronounced in contrary to national guidelines regarding this practice. Most of antibiotics were dispensed without a prescription when the simulator asked any medication to alleviate his/her symptoms.

## 1. Introduction

Antibiotics have mitigated the encumbrance of common infectious diseases and also they become vital for many medical mediations [1]. They are crucial drugs, particularly in the developing world, where infectious diseases are a common cause of death [2]. Furthermore, antibiotics are distinctively considered as societal drugs, since individual usage has a great implication on other individuals in the community and environment [3]. Moreover, antimicrobial resistance is a progressively serious threat to global public health that requires action through all government sectors and society as a whole [3]. Misuse and overuse of antibiotics have driven the emergency of antibiotic-resistant bacteria, which in turn leads to a loss of efficacy of these drugs [4].

Studies have revealed that rates of antibiotic-resistant infections correlate with grades of antibiotic utilization. These inappropriate antibiotics' consumption gives a clue for experts to suspect that the prescription of antibiotics for treating viral infections is part of the problem [5]. Although the increasing resistance can be attributed to several factors, a major cause is the overall volume of antibiotic consumption and especially wrong diagnosis, indiscriminate prescribing, and dispensing errors [6].

Those patients infected with drug-resistant bacteria are at high risk of shoddier clinical outcomes and also they devour more healthcare expenditures than patients infected with nonresistant strains of the same bacteria [7]. According to the study conducted in the European Union, outpatient care costs of infections attributable to antibiotic-resistant bacteria were estimated at about EUR 10 million and productivity losses due to absence from work of infected patients were estimated to be more than EUR 150 million each year. Societal costs of infections due to the selected antibiotic-resistant bacteria in Europe were also estimated to be EUR 1.5 billion each year [8].

The sale of prescription-only medicines without a prescription is an important regulatory issue. Globally, over 50% of antibiotics are sold without a medical prescription. Although over the counter sale of antibiotics is common in the developed world, this practice is more noticeable in developing countries like Ethiopia, where regulation strategies are too weak [9, 10]. A study conducted in Alexandria, Egypt, shows that 65.4% of community pharmacies have sold antibiotics without a prescription [11].

In developing countries, relatively unlimited accessibility and consumption have led to unduly higher incidence of inappropriate use of antimicrobials and greater levels of resistance compared to developed countries due to either wrong medicine or subtherapeutic dose [12]. In all countries, most antimicrobial use occurs outside of hospitals [13]. In developing countries, pharmacies become a primary source of healthcare in the context of limited physician access, and patients often seek their services directly. This makes strict enforcement of regulations on over the counter dispensing of antibiotics challenging [14]. To date, there have been very few studies conducted locally to investigate and file nonprescription sale of antibiotics. The investigators, therefore, aimed to assess the professional practice on OTC sale of antibiotics at community drug retail outlets found in Mizan-Aman town.

## 2. Methods

A cross-sectional simulated client visit study among community drug retail outlets found in Mizan-Aman town, Southwest Ethiopia, was conducted during March 14–28, 2018. Mizan-Aman town is the administrative town of Bench Maji zone which is one of 13 zones of SNNPR. The town is located 582 Km away from Addis Ababa, the capital city of Ethiopia. Currently, there are 18 commercially licensed community drug retail outlets in Mizan-Aman town and the study was undertaken on all drug retail outlets. Also, all drug retail outlets functioning during the data collection period were included in the study.

Data were collected through questionnaire developed by the authors. The questionnaire underwent multiple revisions for its lucidity and compatibility with the purpose of the study. The questionnaire was categorized into three parts: the first part included general information, followed by number of the allocated clinical scenarios and the response of pharmacy professional either by agreeing or by refusing to dispense an antibiotic and the third part included the dialogue that was predicted to occur between the pharmacy professional and the simulator (data collector) regarding the simulated clinical case.

In general, if the pharmacy professional dispensed an antibiotic, the data collector recorded the level of demand (first, second, or third) in which the antibiotic was given and the type of antibiotic. Moreover, the data collector also documented the following information: whether/not the pharmacy professional explained how to take it, specified the duration of treatment, asked about any drug allergies, other symptoms, concomitant use of other drugs, or the pregnancy status of the female client and recommendation to consult a physician. On the other hand, if a pharmacy professional refused to dispense an antibiotic, the data collector was expected to reveal the possible reason for denial.

Three different clinical scenarios were chosen: sore throat, acute diarrhea, and urinary tract infection (UTI) without complications in childbearing-aged women. In each of the three cases, three levels of demand were used to obtain the antibiotic. Initially, the pharmacy professionals were asked if something could be given to relieve the symptoms of the disease (first level of demand). If the antibiotic was not given, the actor used the second level of demand: “This medication is not very strong, can't you give me something stronger?” If the pharmacy professionals still did not provide the antibiotic, the actor openly stated, “I would like an antibiotic,” which was considered the third grade of demand.

If they refused to sell an antibiotic, the reasons for the rejection were requested. A response was considered to be administrative if the reason given only referred to the regulations or law (i.e., the antibiotic cannot be sold without a prescription). Conversely, a reply might be health-related if the pharmacy professionals expressed the following apprehensions: it was not good for the patient's health, an antibiotic cannot be given for viral infections, or selling an antibiotic in this scenario could lead to the spread of resistance.

In an attempt to obtain an antibiotic, simulators pursued pharmacy professionals by three levels of demand:

*Level 1*: Asking something to alleviate his/her symptoms

*Level 2*: Asking for a stronger medication

*Level 3*: A clear request for an antibiotic in the case of not achieving the previous two levels of demand.

The data was collected by three clinical pharmacists. Our study involved three principal investigators/simulators. Each drug store was visited once by investigators who simulated inflicting clinical scenario according to simulated client method pharmacy surveys. Data collectors were trained on data collection procedure and, in order to standardize the information delivered to the pharmacists, a number of rehearsal meetings were accompanied. Additionally, collected data were compiled and presented as descriptive statistics. Lastly, for simulated client method, ruse and incomplete disclosure to study subjects were considered ethically acceptable, since this study was a minimal risk study and it could not have been performed with complete disclosure of the investigator's real entity. Data was kept confidential and anonymous.

### 2.1. Operational Definitions and Definition of Terms

*Antibiotic-Resistance*. It is where new emerging strains of microorganism like bacteria have been found to survive traditional antibiotic exposure or the bacteria are no longer susceptible to an antimicrobial drug.

*Drug Store*. It is a retail store where medicines and miscellaneous articles are sold.

*The Sore Throat Scenario*. There was a 24-year-old male relative experiencing pain in throat with difficulty of swallowing and slight fever for the last 24 hours.

*The Acute Diarrhea Scenario*. There was a 23-year-old healthy male relative experiencing a loose bowel motion accompanied by 4-5 episodes of diarrhea and slight weakness with slight fever for one-day duration.

*The UTI Scenario*. There was a 24-year-old female relative experiencing a burning sensation upon urination for 3 consecutive days.

## 3. Results

The study was carried out on a total of 18 drug stores. Clinical scenarios were equally allocated among the different drug stores included in this study. The majority, 14(78%), of dispensers were males.

### 3.1. Antibiotics Dispensing Practice

Most, 17 (94.4%), of drug stores out of the total 18 in which all three clinical scenarios were allotted were issued antibiotics without need of medical prescription with three different levels of demand. The highest percentage of antibiotic dispensing was associated with the UTI simulation. Antibiotics were sold without a prescription in most, 17 (94.4%), of 18 drug stores in which a urinary tract infection clinical scenario was presented. This was the highest percentage of sales among the 3 clinical scenarios analyzed in this study. Accordingly, 16 pharmacy professionals were issued the antibiotics under the first level of demand, while merely one druggist sold the antibiotics when more strong agents were asked. Similarly, antibiotics were obtained without a prescription for acute diarrhea from 16 (88.9%) drug stores. With respect to sore throat simulation, antimicrobial drugs were obtained without a prescription from 14 (77.8%) drug stores. Majority, 47 (87.2%), of antibiotics dispensed without a prescription were when the simulator asked for any medication to alleviate his/her symptoms. The distribution of the percentage of drug stores that dispense antibiotics without prescription with different levels of demand for the simulated scenarios was summarized in Table 1.

*Level 1* represents the lowest level of demands as the investigator asked for something to alleviate the symptoms.

*Level 2* represents something stronger.

*Level 3* represents the highest level of demand by directly asking the pharmacy professional for an antibiotic.

**Figure 1:**
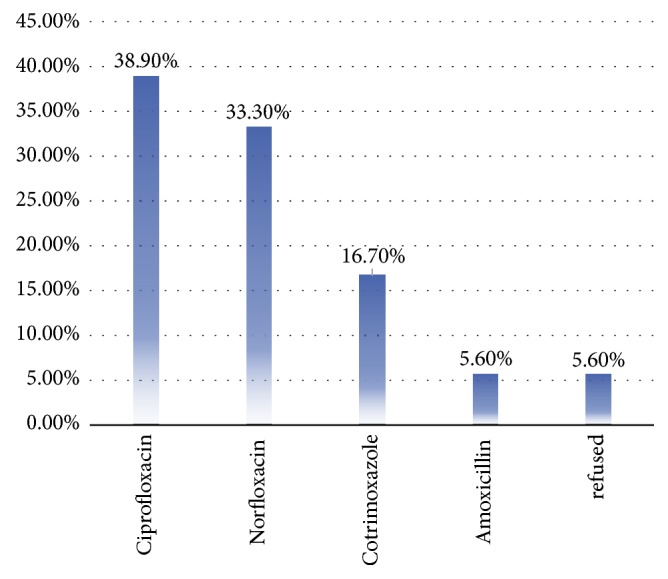
Antibiotics dispensed for UTI clinical scenario at drug store*s* found in Mizan-Aman town, Southwest Ethiopia, March 14-28, 2018.

Ciprofloxacin was the most commonly, 7(38.9%), dispensed antibiotic in cases of the urinary tract infection scenarios followed by Norfloxacin (six drug stores, 33.3%) (Figure 1).

For acute diarrhea, Metronidazole was the most commonly (50 %) prescribed antibiotic in that simulated scenario, whereas Amoxicillin was commonly, 10 (71.4%), given for sore throat scenario (Table 2).

Majority, 9 (64.2%), of the druggists instructed the simulator how to take the antibiotics, while only one dispenser was given a precaution to the investigators about the medication side effects.  Table 3 recaps the main interview aspects and the percentage of dispensed antibiotics across the different case scenarios.

### 3.2. Pharmacy Professional's Additional Inquiry about Simulated Clinical Scenario

Majority of pharmacy professionals asked the simulator for further symptoms when the client was simulated for sore throat (11 [61.1%]), followed by UTI and acute diarrhea (8 (44.4%), each). Conversely, none of pharmacy professionals had asked the simulator about possible drug allergies and concomitant medication usage. Only one (5.56%) pharmacy professional asked the female simulator about her pregnancy status when she was simulated for UTI clinical scenario.

From all drug stores in which the clinical scenarios were presented, eight (44.4%) for acute diarrhea, six (33.3%), and four (22.2%) were not asked for any additional inquiry about presented clinical scenarios (Table 4).

### 3.3. Antibiotic Dispensing Refusal

Out of 18 drug stores, merely a single drug store refused to dispense antibiotics for entirely three simulated clinical scenarios. Regarding each clinical scenario, the majority (22.2%) of this repudiation response came from sore throat cases. Accordingly, two (11.1%) of them were rationalized as their concern related to health issues and antibiotic resistance, whereas one pharmacy professional (5.6%) cited purely administrative reasons for not selling the drug in referring to the prohibition of selling an antibiotic without an official prescription signed by a physician. Also, the remaining one druggist (5.6%) refused to sell antibiotics due to fear of misdiagnosis.

Only one dispenser (5.6%) for UTI scenario and two professionals (11.1%) for acute diarrhea case refused to dispense antibiotics without prescription due to fear of misdiagnosis. In all drug stores in which they refused to dispense antibiotics, they recommended simulator to visit the physician.

## 4. Discussion

Our study revealed over the counter sale of antibiotics at drug stores found in Mizan-Aman town with neither a prescription nor a diagnosis from a physician. The investigators simulated for UTI, sore throat, and acute diarrhea to obtain antibiotics by using various levels of demand. This cross-sectional simulated client visit study has shown that almost all (94.4%) of drug stores sold antibiotics without a prescription. This finding is similar to the study done in Pune, India, by S.d. salunkhe* et al*., in which over the counter sale of antibiotics was 94.56% [15]. Besides, our finding is also comparable with the finding of the study done in Addis Ababa, Ethiopia, by Daniel Asfaw* et al*., which reported a very high rate of dispensing of prescription-only medicines without a prescription [16].

On the other hand, our result is too higher than the finding of Lebanese study, which revealed that 32% of drug retail outlets sold antibiotics without a medical prescription [17]. This discrepancy might be due to differences in study design, since their study design was descriptive, cross-sectional, in which they simply assess pharmacists' attitudes and knowledge about over the counter sale of antibiotics. In contrary, we assessed the actual practical aspect of professionals because we conducted a cross-sectional simulated client visit study. Furthermore, variation in sample size and study participants might also be the possible reasons.

According to present study, regarding each clinical scenario, the percentage of drug stores dispensing antibiotics without a prescription for the urinary tract infection scenario was 94.4%, followed by acute diarrhea (88.9%), and 77.8% was for sore throat scenario. This study is comparable with the study done in Jordan by Ammar Almaaytah* et al*., which showed the percentage of pharmacies dispensing antibiotics without a prescription for the sore throat scenario to be 97.6%, followed by urinary tract infection (83.3%) and diarrhea (83%) [18]. In addition, the finding of study done in Riyadh also shows equivalent results, in which simulated cases of sore throat and diarrhea resulted in an antibiotic being dispensed in 90% of encounters and 75% for UTI [19]. On the other hand, our finding is not in line with study piloted in Catalonia, Spain, in which antibiotics were obtained from 79.7% of pharmacies when a urinary tract infection was simulated and from 34.8% of pharmacies when a sore throat was simulated [20]. This inconsistency might be attributed to variation in study setting and disparities in healthcare systems affecting access to healthcare.

The results of our study clearly demonstrated that antibiotics could be easily acquired and sold without the need to supply a medical prescription to the pharmacy professional. From the clinical scenarios simulated in this study, sore throat is mainly caused by viral cause rather than bacterial pathogen, which did not require antibiotic utilization. Moreover, since acute watery diarrhea is self-limited, antibiotics should not have been prescribed without stool tests to identify the likely pathogen. Therefore, using antibiotics for nonbacterial or viral pathogen will expedite antibiotic-resistance, which is the major potential sequelae associated with this practice. As a result, antibiotic-resistance in turn leads to an extra cost burden to the patient owing to the need for broader-spectrum antibiotics, additional prescriptions, and hospitalizations for antibiotic failures. Dispensing of antibiotics for nonbacterial diseases that actually do not require antibiotics could be terrible [21]. Our study had shown that Fluoroquinolones were the most commonly dispensed antibiotics for presumed UTI in a child-bearing-age woman without verifying the pregnancy status. Consequently, they could subject the fetus to hazardous congenital abnormalities, since quinolones are contraindicated in pregnancy.

In our finding, antibiotics were dispensed without a prescription with three different levels of demand. Most (87.2%) of antibiotics were dispensed without a prescription when the simulator asked for any medication to alleviate his/her symptoms (first level of demands). Only one drug store refused to dispense antibiotics without a prescription with all three level of demands. This finding is almost similar to the study done in Riyadh, Saudi Arabia, which reported that 90% were dispensed with first level demands and only one drug store denied dispensing with all three-level requests [19]. On the contrary, the study done in Jordan reported that 59.2% of antibiotics were obtained by first level of demands [18]. This disparity might be due to difference in study setup and sample size.

Obtaining antibiotics without a prescription will not only promote antimicrobials resistance but can also be associated with significant adverse events including drug adverse effects, high cost, and complications. According to our study, none of the pharmacy professionals asked about prior history of drug allergy and concomitant drug use.

## 5. Limitation of the Study

Sample size considered was small. Additionally, since three levels of demand were used consecutively until an antibiotic was bestowed, the simulators claimed obtaining an antibiotic when the pharmacy professional denied so as to show the effect of perseverance on obtaining an antibiotic when it is not clearly required. We were aware of the fact that this technique could have augmented the number of offers of antibiotics by the dispensers.

## 6. Conclusion

The results of this study demonstrate that nonprescription sales of antibiotics were highly pronounced in contrary to national guidelines regarding this practice. Commonly dispensed antibiotics were Amoxicillin, Metronidazole, and Ciprofloxacin to treat complaints of sore throat, acute diarrhea, and UTI, respectively. Most of antibiotics were dispensed without a prescription when the simulator asked for any medication to alleviate his/her symptoms.

## Figures and Tables

**Table 1:** Pharmacy professionals' antibiotics dispensing practice without a prescription with regard to level of demand at drug stores found in Mizan-Aman town, Southwest Ethiopia, March 14-28, 2018.

Level of demand	Sore throat n=14	UTI n=17	Acute diarrhea n=16
Level 1	14(100%)	16(94.1%)	11(68.75%)
Level 2	0 (0%)	1(5.9%)	3(18.75%)
Level 3	0 (0%)	0 (0%)	2(12.5%)

**Table 2:** Antibiotics dispensed for acute diarrhea and sore throat clinical scenarios at drug stores found in Mizan-Aman town, Southwest Ethiopia, March 14-28, 2018.

Clinical scenarios	Antibiotics	Frequency (%)
Acute diarrhea	Metronidazole	8 (50.0%)
(N=16)	Rifampicin	4(25.0%)
	Tinidazole	2(12.5%)
	Ciprofloxacillin + Tinidazole + Metronidazole	2(12.5%)
Sore throat	Amoxicillin	10(71.4%)
(N=14)	Azithromycin	3(21.4%)
	Amoxicillin + Azithromycin	1(7.2%)

**Table 3:** Pharmacy professional counseling and explanation to simulated clinical scenarios at drug stores found in Mizan-Aman town, Southwest Ethiopia, March 14-28, 2018.

Pharmacy professional Statements	Number (%) of drug stores providing antibiotics		
	Clinical case presented		
	Sore throat (n= 14)	UTI (n=17)	Acute diarrhea (n=16)
Only explained how to take the antibiotics	9(64.3%)	15(88.2%)	5(31.2%)
Explained both how to take the antibiotics and duration of treatment	1(7.1%)	-	4(25%)
Explained only instruction on side effects of dispensed antibiotic(s)	1(7.1%)	1(5.9%)	1(6.2%)
Providing no counseling regarding dispensed antibiotics	3(21.4%)	1(5.9%)	3(18.7%)
Other(s)_ _^*∗*^	-	-	3(18.7%)

**∗**Explained how to take the antibiotics as well as duration and side effects and explained how to take the antibiotics and side effects.

**Table 4:** Pharmacy professionals' additional inquiry about simulated clinical scenario at drug stores found in Mizan-Aman town, Southwest Ethiopia, March 14-28, 2018.

Pharmacy professional inquiry	Simulated clinical scenarios		
	Sore throat	UTI	Acute diarrhea
Only asked for further symptoms	11(61.1%)	8(44.4%)	8(44.4%)
Asked about prior drug allergy	0(0%)	0(0%)	0(0%)
Asked only concomitant use of another drug	0(0%)	0(0%)	0(0%)
Asked pregnancy status of the female simulator for UTI	-	1(5.56%)	-
Other*∗*	1(5.56%)	1(5.56)	2(11.1%)
No additional inquiry	6 (33.3%)	8 (44.4%)	8(44.4%)

**∗**Asked further symptoms and about environmentalsanitation, further symptoms, and dose.

## Data Availability

The supporting documents for this study can be available from the corresponding author upon request.

## Abbreviations

AMA: Antimicrobial
OTC: Over the counter
SNNPR: Southern Nations, Nationalities, and Peoples' Region
SPSS: Statistical Package for Social Sciences
UTI: Urinary tract infection
WHO: World Health Organization.
